# Adapting a neuroscience-informed intervention to alter reward mechanisms of anorexia nervosa: a novel direction for future research

**DOI:** 10.1186/s40337-021-00417-5

**Published:** 2021-05-26

**Authors:** Ann F. Haynos, Lisa M. Anderson, Autumn J. Askew, Michelle G. Craske, Carol B. Peterson

**Affiliations:** 1 Department of Psychiatry and Behavioral Sciences, University of Minnesota, 2450 Riverside Ave., Minneapolis, MN 55454 USA; 2 Department of Psychology and Department of Psychiatry and Biobehavioral Sciences, University of California, Los Angeles, Los Angeles, CA USA

**Keywords:** Anorexia nervosa, Positive affect, Reward, Treatment, Intervention, Neuroscience

## Abstract

Accumulating psychobiological data implicate reward disturbances in the persistence of anorexia nervosa (AN). Evidence suggests that individuals with AN demonstrate decision-making deficits similar to those with mood and anxiety disorders that cause them to under-respond to many conventionally rewarding experiences (e.g., eating, interacting socially). In contrast, unlike individuals with other psychiatric disorders, individuals with AN simultaneously over-respond to rewards associated with eating-disorder behaviors (e.g., restrictive eating, exercising). This pattern of reward processing likely perpetuates eating-disorder symptoms, as the rewards derived from eating-disorder behaviors provide temporary relief from the anhedonia associated with limited responsivity to other rewards. Positive Affect Treatment (PAT) is a cognitive-behavioral intervention designed to target reward deficits that contribute to anhedonia in mood and anxiety disorders, including problems with reward anticipation, experiencing, and learning. PAT has been found to promote reward responsivity and clinical improvement in mood and anxiety disorders. This manuscript will: (1) present empirical evidence supporting the promise of PAT as an intervention for AN; (2) highlight nuances in the maintaining processes of AN that necessitate adaptations of PAT for this population; and (3) suggest future directions in research on PAT and other reward-based treatments that aim to enhance clinical outcomes for AN.

## Plain English summary

Past research has shown that people with anorexia nervosa (AN) have trouble experiencing positive emotions in response to many common life events (e.g., socializing, winning money). This tendency to under-respond to positive events is similar to that found in people with mood and anxiety disorders. However, people with AN also appear to feel more positive emotions when they think about or engage in eating-disorder behaviors (e.g., cutting back on their eating, exercising, thinking about being very thin). For this reason, people with AN may continually turn to eating-disorder behaviors, rather than other actions, to feel good about themselves and their lives. In this paper, we describe a new treatment, Positive Affect Treatment (PAT), that we believe has the potential to help individuals with AN gain more happiness and fulfillment from their lives outside of their eating disorder. Our expectation is that such a change may result in individuals with AN no longer needing their eating disorder to feel good.

## Introduction

Anorexia nervosa (AN) is a psychiatric disorder associated with serious physiological and psychological morbidity, increased risk of mortality, and, for some, enduring illness courses [1–3]. One empirically-supported treatment has emerged with the strongest efficacy for treating adolescent AN [4]. However, despite recent growth in efficacious treatments for adult AN, no existing intervention has emerged clearly superior over others [5]. As such, there have been calls for increased efforts to develop innovative interventions for this population that may more precisely target the maintenance mechanisms of this disorder [6]. Indeed, one potential barrier to effective treatment for AN is that many treatments to date have been limited in their incorporation of the field’s growing knowledge on the maintaining mechanisms of AN [6, 7].

Several theories have suggested that aberrations in the experience of positive affect may perpetuate the symptoms of AN [8, 9]. These reward-based theories have been bolstered by an expansion of neuroimaging research that demonstrates abnormalities in the ways in which the brain processes reward in AN [10]. Despite a preponderance of evidence suggesting that problems with processing reward and positive affect play a critical role in the symptomatology of AN, no treatments for this population have been designed that designate increasing positive affect as a primary intervention target.

Here we briefly review evidence that reward processes present viable targets for AN treatment. We then outline and describe one potential approach for targeting reward abnormalities in AN: Positive Affect Treatment (PAT), a novel psychotherapy intervention that has been used to alter positive affect and reward processes in anxiety and mood disorders [11]. We suggest a proposed adaptation of this treatment for AN that we are currently testing (PAT-AN), and highlight potential avenues for future research. In addition to describing a promising treatment for AN, this manuscript highlights the need to consider positive affect as a critical target in interventions for AN.

## Evidence for reward disturbances in AN

Many have comprehensively reviewed the reward literature in AN [8–10, 12–14]. Below we provide a selective overview of the literature in AN relevant to specific reward targets that have been determined to influence other mental health concerns [15], and to serve as appropriate treatment targets in psychiatric populations [11]. Reward system dysregulation has been implicated in a number of psychiatric disorders [16]. Prominent neurobiologically-informed reward theories suggest that reward processing can be decomposed into three stages that occur before, during, and after reward receipt: reward anticipation (or “wanting”), experiencing (or “liking”), and learning [15, 17]. Reward anticipation refers to positive expectancy, desire, or effort for a future pleasant experience. Reward experiencing is the ability to derive pleasure from rewards once they are obtained. Reward learning reflects the ability to internalize the information provided by rewards and to use it to guide future approach behavior. A deficit in any one of these stages of the reward process could result in low positive affect or an inability to direct behavior towards valued life goals. Existing research indicates that individuals with psychiatric disorders vary on the degree to which reward anticipation, experience, or learning are impacted [18]. For instance, each of these reward stages appears to be impaired in depressive disorders [15, 18], but reward anticipation deficits appear to feature most prominently in schizophrenia [18].

Thus, we will review the evidence on the functioning of reward anticipation, experiencing, and learning in AN. Additionally, we will review the literature on disorder-specific reward processes (heightened reward anticipation, experiencing, and learning related to weight-loss cues) that differentiate AN from other disorders.

## Reward anticipation

On self-report temperament measures, individuals with AN score lower on sensation-seeking measures, which capture drive to seek novel hedonic rewards, compared to healthy comparison (HC) and other eating-disorder groups [19]. Further, compared to HCs, individuals with AN demonstrate lower implicit and explicit anticipation of reward from palatable foods [20, 21] and interpersonal experiences, such as prosocial touch [22]. Individuals with AN, especially restricting subtype, also exhibit greater preference for delayed over immediate monetary gains compared to HCs and individuals with bulimia nervosa or binge eating disorder [23]. This pattern of delay discounting findings has been commonly attributed to decreased desire for immediate rewards, or increased engagement of cognitive control over immediate rewards [10].

Activity in particular brain regions, especially the ventral tegmental area, amygdala, and ventral striatum, has been associated with reward anticipation [15, 17]. There evidence of aberrant neural responding during anticipation of monetary rewards in AN. Some studies have found that individuals with AN over-engage brain regions promoting cognitive control and punishment, such as the dorsolateral prefrontal cortex and insula, during reward anticipation [24, 25]. This over-engagement of control- and punishment-related brain regions could indicate that the expectation of a positive event evokes fear or desire to exert top-down control over emotions. This hypothesis corresponds with data identifying that individuals with AN avoid positive, in addition to negative, emotional experiences [26]. Indeed, reducing prefrontal cortex activity (and, thereby, cognitive control) during reward anticipation has been associated with greater weight gain during treatment [24]. Individuals with AN also display less activation of brain regions traditionally linked to reward anticipation, such as the striatum, when delaying monetary receipt [27]; thus, expectation of immediate gratification may be less appealing in this group.

## Reward experiencing

Research using implicit and explicit measures has shown that typically rewarding experiences, such as viewing or consuming palatable foods [20, 21, 28, 29] watching humorous videos [30, 31], or perceiving prosocial emotions or touch [22, 32, 33] are less subjectively pleasant, and in some cases more aversive, for individuals with AN compared to HCs. Reward experiencing has been shown to engage similar brain structures as reward anticipation, most notably the ventral tegmental area and striatum interacting with the orbitofrontal cortex [15, 17]. Individuals with AN exhibit less activation in reward-related brain regions (e.g., ventral tegmental area, dorsal striatum) in response to palatable food cues compared to those with bulimia nervosa [21, 34]. Paralleling the literature on reward anticipation, individuals with AN also show greater activation of cognitive control circuitry (e.g., medial or dorsolateral prefrontal cortex) during the receipt of food [35–37], social [38], and monetary [25] rewards compared to HC participants. Further, while hunger enhances reward experiencing among HC participants, reflecting a biological tendency to seek rewards (such as food) in a deprived state, reward-related brain activation is unaffected by hunger in AN [39]. Further, amphetamine-stimulated dopamine release in the ventral striatum, which is experienced by most as euphoric, is experienced instead as anxiogenic among individuals with AN [40]. The anxiogenic quality of dopamine release may explain why individuals with AN find immediate, high-intensity, and uncontrollable rewarding stimuli less enjoyable [10].

## Reward learning

Individuals with AN have been found to perform poorly on decision-making games that require them to learn to obtain rewards based on feedback [41]. Individuals with AN tend to demonstrate inflexible decision-making on these and other decision tasks; once rewarded, their responses persist without incorporating new and changing reward information [42]. The bias towards previously-rewarded stimuli parallels the presentation of AN, in which weight-loss behaviors persist well beyond the point at which they no longer garner reward [7].

Reward learning is most typically associated with activation among varied regions of the prefrontal cortex that process signals received from subcortical reward structures [15, 17]. There is evidence that individuals with AN demonstrate less differentiation between rewards and losses in the ventral striatum during reward learning tasks, suggesting a possible disruption of signal from this region interrupting consolidation of learning in the prefrontal cortex [43]. Similar findings pertain to prediction error (the discrepancy between expected and actual outcomes), which can be viewed as a learning signal, in AN. Greater prediction error (surprise at an unexpectedly good or bad outcome) typically leads to greater adjustment to future expectations. However, individuals with AN show abnormal prediction error responses to unexpected losses and gains of general stimuli (money) and disorder-specific stimuli (food) characterized by greater responsivity in punishment-related regions (e.g., insula) [24, 44]. Thus, findings relating to predictor error may reflect a tendency to prioritize punishment information over reward information in making future decisions.

## Reward from weight loss cues and behaviors

In contrast to the above research on processing of disorder-irrelevant rewards, there is evidence for *elevated* reward anticipation, experiencing, and learning specifically in the context of weight loss in AN. This cue-specific reward responsivity differentiates AN from other psychiatric disorders (e.g., mood and anxiety disorders), which show deficit reward processing across contexts [15]. In AN, drive and activity for reward is enhanced in anticipation of weight-loss behaviors, such as restrictive eating and exercise [45, 46]. Additionally, individuals with AN show enhanced implicit and explicit reward experiencing in response to weight-loss cues, such as low-calories foods [20, 47, 48], exercise stimuli [49], and underweight bodies [50, 51]. Similarly, ecological momentary data collected from individuals with AN in real-time have found that positive affect is elevated during restrictive eating and exercise episodes [52, 53]. Self-conscious positive affect (e.g., pride) appears to be an especially important class of reinforcers maintaining eating-disorder behavior in AN. Studies have found that self-assurance increases in anticipation of exercise and following restrictive eating episodes in AN samples [53, 54] and that enhancing pride is cited as one of the primary motivators for restrictive eating among individuals with eating disorders, including AN [55].

There is also initial evidence for neural underpinnings of enhanced disorder-relevant reward in AN. Compared to HCs, individuals with AN demonstrate increased activity in the ventral striatum in response to pictures of underweight bodies [50, 56]. Further, activation and connectivity in cognitive control regions is decreased in response to low-calorie food and exercise images in AN [47, 57], suggesting that reward responding to these disorder-specific stimuli, in contrast to other rewards, is less constrained by cognitive control.

## Summary and theoretical model

Theories developed through the investigation of reward-based behavior and brain activity suggest that reward anticipation, experiencing, and learning in AN are often diminished in response to certain situations (i.e., those involving food, social interaction, and monetary rewards). Further, there are data highlighting neurobiological disturbances in the functioning of reward-related neural circuitry in AN. There is also evidence of a complex interplay between cognitive control mechanisms and reward in AN, which may reflect cognitive barriers impeding the ability to expect or experience reward. Paradoxically, rewards related to AN symptoms (i.e., weight loss) are highly appealing to this population.

**Fig. 1:**
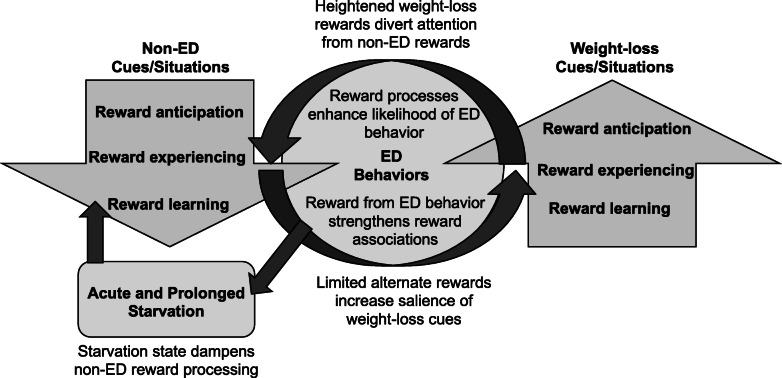
Transactional Theoretical Model of Reward Processing in Positive Affect Treatment for Anorexia Nervosa (PAT-AN). Note: ED = Eating Disorder

As highlighted in Fig. 1, transactions between low levels of positive affect derived from typically rewarding experiences (unrelated to the eating disorder) and elevated positive affect from weight-loss cues may maintain eating-disorder symptoms over time. Limited access to reward from most positive experiences (due to low trait reward responsivity or cognitive control applied too frequently or inflexibly) can generate an aversive anhedonic state. In this case, if reward responding is heightened to weight loss and related cues, the resulting momentary positive affect enhancement could serve as a powerful instrumental reinforcer, thus strengthening eating-disorder symptoms. The more an individual engages in eating-disorder behavior and receives a subsequent reward, the more likely an individual will be behaviorally and biologically compelled to seek out disorder-related, as opposed to disorder-unrelated, rewards. Thus, over time, disrupted reward processing may increase the desire to engage in eating-disorder behavior, and eating-disorder behavior may enhance reward system abnormalities. Further, as starvation is prolonged and individuals become underweight, reward responding becomes biologically dampened to all stimuli [58], potentially enhancing the need to rely on the most compelling or practiced reward-eliciting experiences (e.g., weight-loss behaviors) to feel good. This cycle presents several critical targets that have not yet been addressed in AN treatment.

## Positive affect treatment: a potential approach to treating reward disturbances in AN

PAT is a treatment that has potential to impact reward abnormalities in AN. PAT is a cognitive-behavioral intervention designed by for the treatment of anhedonia in mood and anxiety disorders [11, 15]. Based on an affective neuroscience model, PAT aims to increase positive affect by engaging the appetitive “approach” system in contrast to the withdrawal “defensive” system that functions to avoid negative outcomes. A primary rationale for the development of PAT was the modest outcomes among existing treatments for depression and anxiety that more typically focus on reducing negative emotions [15]. PAT was specifically developed to target aspects of neurobiological reward processing outlined above: reward anticipation, experiencing, and learning [11]. In a randomized trial in which PAT was compared with a cognitive behaviorally-based negative affect treatment targeting threat sensitivity, PAT was more efficacious in increasing positive emotions and reducing negative emotions, depression, anxiety, stress, and suicidal ideation among individuals with depressive and anxiety disorders [11]. Other trials of this treatment are ongoing (ClinicalTrials.gov: NCT03439748).

The rationale for adapting PAT for AN is based on the above-described research demonstrating neurobiological-based reward and positive affect abnormalities in AN, which suggest that a treatment directly targeting positive affect may be well suited for this population. Below we provide an overview of the components and interventions included in PAT and the proposed adaptations that we suggest to accommodate the symptom presentation and presumed mechanisms of AN. These adaptations are currently being evaluated in an ongoing pilot study (ClinicalTrials.gov: NCT04007900). However, it is important to note that, at this point, the proposed adaptations constitute an idea warranting further investigation, rather than an empirically-supported treatment for AN. Although the scientific basis for PAT-AN is not yet established, the description of this treatment in this manuscript is intended as a launching point for consideration of potential applications of PAT (or alternate reward-based treatments) for AN. As such, we differentiate components that have been empirically tested in mood and anxiety disorders (Standard PAT) and the proposed adaptations to the PAT manual that we are currently testing in AN, for which empirical evidence is forthcoming (Proposed Adaptions for AN).

## Overarching proposed adaptions for AN

In our pilot study, we have made three overarching changes to the PAT manual intended to support its application to AN. First, consistent with the original PAT manual [11], our adapted PAT-AN aims to increase positive emotions by using interventions to target global reward anticipation, experiencing, and learning. However, based on the above-described theoretical model (Fig. 1), we have adjusted PAT-AN also to emphasize reducing positive affect and reward associated with eating-disorder symptoms and replacing positive eating-disorder experiences with rewards derived independent of the eating disorder. Thus, the primary targets of treatment are considered: 1) to increase positive affect outside of eating-disorder symptoms; and 2) to decrease positive affect derived from eating-disorder behavior. To facilitate this adaptation to treatment goals, we have added an eating-disorder-specific module to the treatment. Although weight gain is expected and encouraged, it is hypothesized to result as a product of the primary treatment aims, and is not currently targeted separately from positive affect processes.

Second, to accommodate the additional eating-disorder module and to match PAT-AN to more standard eating-disorder treatment durations [59, 60], we extended PAT-AN from 16 [11] to 20 individual psychotherapy sessions, currently delivered in an outpatient setting. Thus, our proposed adaptation of PAT involves six, rather than the original five [11], therapy modules: Psychoeducation (Module 1), Pleasant Events Scheduling (Module 2), Attending to the Positive (Module 3), Cultivating the Positive (Module 4), Replacing Positive Aspects of the Eating Disorder (Module 5); and Relapse Prevention (Module 6) (see Table 1). In the ongoing pilot study of PAT-AN, participants have been permitted to pursue other concurrent treatments and PAT-AN has been delivered either in a standalone or adjunctive manner.

**Table 1:** Overview of Positive Affect Treatment (PAT) for Anorexia Nervosa (AN)

**Module 1: Psychoeducation (Session 1)**		
**Activity**	**Proposed Adaptations for AN**	**Target**
1. Treatment rationale: learning about treatment content, structure, goals, and background	Sharing AN-specific model; discussing how weight and eating goals will be integrated into primary goal of increasing non-eating-disorder-related positive affect	Education
2. Introducing positive mood: learning about positive mood, its importance, and its difference from negative mood	Considering which positive emotions are most closely linked to different eating-disorder behaviors	Education
3. Parts of mood and the mood cycle: learning about the components of mood and how these different parts can cause low positive mood	Discussing the impact of eating-disorder behaviors, starvation, and underweight on positive mood	Education
**Module 2: Pleasant Events Scheduling (Sessions 2–7)**		
1. Activity planning: identifying events to engage enjoyment, mastery, and/or values	Selecting non-eating-disorder activities that may share a similar function to eating-disorder symptoms; considering how to use exercise as a healthy, rather than disordered, reward (as needed); prioritizing social and long-term rewards; evaluating values	Reward anticipation
2. Activity engagement: executing planned events; recording event-reward associations	Recording link between positive affect, pleasant events, and eating-disorder symptoms; noticing cognitive barriers to positive affect	Reward experiencing and learning
3. Activity recounting: learning and practicing techniques to “savor” past pleasant experiences	Sharing pre-recorded exercise to provide a concrete script; option for socially “co-savoring” if struggling with experiential exercise	Reward experiencing
**Module 3: Attending to the Positive (Sessions 8–11)**		
1. Finding the silver lining: training to shift attention to positive aspects of daily life and positive stimuli	Noticing the silver lining of difficult aspects of recovery	Reward anticipation
2. Taking ownership: identifying associations between one’s own behavior and rewards	Emphasizing the potential importance of taking ownership to increase a sense of self-control and agency in life; identifying and appreciating one’s own behavior towards recovery	Reward experiencing and learning
3. Imagining the positive: learning to attend to positive future events	Sharing pre-recorded exercise to provide a concrete script; focusing on goals that will be facilitated through recovery	Reward experiencing and learning
**Module 4: Cultivating the Positive (Sessions 12–15)**		
1. Loving-kindness and appreciative joy: practicing mental acts of giving	Sharing pre-recorded exercise to provide a concrete script; increasing focus on love, kindness, and appreciation towards oneself to potentially reduce the need for eating-disorder behaviors to boost positive self-referential feelings	Reward experiencing
2. Generosity practice: practicing physical acts of giving	Noting the importance of balance of generosity towards others and towards oneself; increasing focus on generosity towards oneself to potentially reduce the need for eating-disorder behaviors to boost positive self-referential feelings	Reward experiencing
2. Gratitude practice: fostering the ability to appreciate the positive aspects of life	Noticing positive aspects of recovery	Reward experiencing
**Module 5: Replacing Positive Aspects of the Eating Disorder (Sessions 16–19)**		
1. Monitoring and replacing eating-disorder behavior: diverting attention away from eating-disorder behaviors to healthier alternatives that can elicit positive mood	Skill specific to proposed AN adaptation of PAT	Reward learning
2. Riding the roller coaster: delaying acting on eating-disorder behaviors when positive emotion is low	Skill specific to AN adaptation of PAT	Reward anticipation and learning
3. Counter-conditioning: incorporating positive experiences into situations associated with negative mood, especially surrounding eating-disorder triggers	Skill specific to proposed AN adaptation of PAT	Reward experiencing and learning
4. Breaking the links: removing environmental cues that are associated with positive emotions related to the eating disorder	Skill specific to proposed AN adaptation of PAT	Reward anticipation and learning
**Module 6: Relapse Prevention (Session 20)**		
1. Check-in: assessing mental health and brainstorming ideas about how to increase the experience of positive emotions	Evaluating progress on weight and eating restoration, as well as cognitive eating-disorder thoughts; planning for future eating-disorder lapses and relapses	Reward experiencing and learning

Note: Adapted from Craske et al., personal communication

Third, due to the established difficulties accessing reward from social experiences in AN [22, 32, 33], we have proposed an adaptation in PAT-AN to explicitly use the therapeutic relationship as an opportunity to build connections between interpersonal relationships and positive affect. The hypothesis is that a therapeutic stance that is consistently non-judgmental, validating, and strengths-based may establish therapist appreciation of the patient’s progress as a motivator for future behavior change [61].

## Module 1: Psychoeducation

### Standard PAT

PAT begins with an introductory session to establish the treatment rationale and expectations. Patients are provided information about the origin of PAT as a treatment for anxiety and depression, including an overview of initial treatment results in these populations [11]. The treatment introduction also provides psychoeducation about the different components of positive affect targeted in treatment and how increasing positive affect is distinct from decreasing negative affect. As in other treatments [59], an emphasis is placed on making refinements to the model according to the patient’s experiences. If a patient is skeptical about the model fit with their personal experience, they are invited to “test” the model throughout treatment by noticing how positive affect affects their life and behavior. The importance of skill practice is emphasized and a workbook is provided to guide homework assignments.

### Proposed adaptions for AN

In the pilot adaptation of AN, patients are also described how PAT has been adapted for AN to additionally target putative disorder-specific positive emotions. The proposed theoretical model (Fig. 1) is presented and discussed with the patient, emphasizing that individuals with AN may be prone to low positive affect for many reasons, including trait differences in how their brains process rewards and the impact of malnourishment [10]. The therapist highlights that weight-loss behaviors (e.g., exercise, purging) may lead to short-term increases in positive emotions, possibly contributing to the maintenance of AN, but also long-term decreases in positive emotion [58]. Therefore, they are informed that treatment will emphasize identifying other sources of positive emotion that do not involve engaging in eating-disorder behaviors. The importance of increasing cognitive flexibility around rewards is especially highlighted to individuals with AN, who appear to demonstrate enhanced cognitive control and rigidity surrounding rewards [24, 25, 35–38].

Patients are informed that, although weight and eating-disorder behaviors currently are not targeted as directly in PAT-AN as in other interventions (e.g., through monitoring food logs [59]), normalizing weight and eating are expected goals of treatment. Patients are given the expectation that they will be weighed weekly and that weight patterns will inform intervention (i.e., using the skills to increase weight if it decreases). Patients and therapists collaboratively decide how much the patient should know of their weight depending on the impact of this information upon positive affect. For instance, if the patient believes that learning about weight loss would making them feel good, reinforcing the presumed link between positive affect and eating-disorder symptoms, weighing may be blind and weight change discussed in terms of general patterns. In the current iteration of treatment, there is no formal monitoring of eating behavior built into treatment. However, the patient is informed that the therapist will check in on eating-disorder symptoms briefly at the beginning of each session and adapt skills for use in reducing reliance on eating-disorder behaviors.

## Module 2: pleasant event scheduling

### Standard PAT

Referred to as “augmented behavioral activation” [11], PAT Module 2 focuses on increasing behaviors that elicit positive affect. Originally described by Lewinsohn [62] the premise of pleasant events scheduling is that avoidance, disengagement, and rigid adherence to routine perpetuates low positive affect. Thus, PAT aims to identify and schedule new activities that elicit positive affect and then monitor the impact on mood. For many patients, small incremental goals are essential to build motivational momentum. Therefore, the variety and novelty of positive affect-eliciting activities gradually increases over treatment. Most critically, behavioral activation is followed by intensive savoring of positive activities through imaginal recounting. In and between sessions, patients are guided to recount the most positive moments of a pleasant activity, including a detailed re-imagining of the sensory and emotional experience, to enhance reward experiencing and learning.

### Proposed adaptions for AN

In line with our proposed model of reward processing in AN (Fig. 1), the preliminary adaptation of Module 2 for AN currently focuses on identifying rewarding activities that are independent of or inconsistent with eating-disorder symptoms, especially those that elicit similar positive emotion states (e.g., pride, safety) as the eating-disorder behaviors of interest [55]. Under conditions in which positive affect has been closely linked with eating-disorder behaviors over time, values clarification exercises may be useful in identifying alternate important and fulfilling activities [63]. Because pride, self-control, and long-term rewards are so frequently linked to weight-loss behavior in AN [23, 53–55], we suggest that engaging in other non-eating-disorder activities that involve long-term planning or accomplishment may be especially fruitful for this population. Additionally, because of the noted deficits in social reward experiencing in AN [10], we surmise that goals involving social contact also may be important to encourage. The current manual also provides suggestions for adapting experiential exercise of pleasant event “savoring” to better suit the concrete thinking style characteristic of AN (e.g., re-experiencing in the form of a social conversation, rather than as an imaginal experience) [64].

## Module 3: attending to the positive (cognitive training)

### Standard PAT

PAT Module 3 emphasizes understanding and altering negative cognitive biases that can perpetuate low positive affect, particularly the tendency to focus only on negative stimuli without noticing neutral and positive stimuli in a situation [11]. In the “Silver Lining” exercise, for example, patients identify positive aspects of experiences initially considered negative. The process of focusing on positive stimuli increases both positive emotion and cognitive flexibility. Through the “Taking Ownership” skill, patients also practice identifying ways that they personally contribute to positive events to increase self-efficacy and reduce negative attributional bias. Additionally, Module 3 includes exercises to practice reward anticipation, in which patients repeatedly focus on imagining specific positive details of an upcoming positive event [65].

### Proposed adaptions for AN

Given the evidence suggesting heightened or rigid cognitive control over reward in AN [24, 25, 35–38], increasing cognitive flexibility to enhance positive affect is likely to be an especially important skill in this population. Additionally, because self-conscious positive emotions have been especially linked to eating-disorder behaviors in AN [23, 53–55], the “Taking Ownership” skill may be particularly important to emphasize in order to build a sense of agency disconnected from eating-disorder symptoms. Further, in our current adaptation of PAT-AN, we have attempted to tie the exercises in this module to eating-disorder recovery as often as possible, for example, practicing finding the “silver lining” in eating a difficult meal or taking ownership of progress towards weight restoration.

## Module 4: cultivating the positive (compassion training)

### Standard PAT

Module 4 includes mindfulness-based exercises that increase reward experiencing [11], including loving kindness, generosity, gratitude, and appreciation practices [66–68]. In the loving kindness meditation, for example, the patient focuses on wishing happiness, safety, and peace towards others and themselves. In gratitude practice, the patient identifies and records things for which they feel grateful daily.

### Proposed adaptions for AN

For individuals with AN, the association between eating-disorder symptoms and self-conscious emotions [23, 53–55] suggests that a self-focus in loving kindness, generosity, and gratitude exercises may be especially impactful in replacing the reward functions of eating-disorder behavior. However, it is possible that self-compassion-focused exercises would be challenging if individuals have relied on eating-disorder behaviors to generate positive self-directed feelings. Thus, in the current iteration of PAT-AN, we provide examples of how to support patients who find self-compassion exercises challenging, such as allowing the patient to generate compassion for themselves in the past (e.g., as a child) or future (e.g., following recovery). As with other experiential exercises, we surmise that making the mindfulness-based exercises in this module as concrete as possible (e.g., with an audio recording) may be helpful for adapting to the cognitive style of many patients with AN [64].

## Module 5: replacing positive aspects of the eating disorder

### Proposed adaptions for AN

Module 5 was added specifically for the version of PAT-AN under investigation. As such, the utility of this module in the treatment of AN has not yet been determined. In this module, the goal is to decrease the experience of positive emotion associated with eating-disorder symptoms and to shift these positive emotions to contexts independent of the eating disorder, consistent with our theoretical model (Fig. 1**)**. As such, many exercises have been drawn from established therapies that target other positively reinforced problematic patterns (e.g., drug abuse, non-suicidal self-injury) [69, 70]. The module starts with self-monitoring the antecedents and consequences of eating-disorder behavior to determine how eating-disorder behaviors may function to elicit positive affect (e.g., exercise leading to accomplishment). After monitoring, the first goal is to increase and differentially reinforce alternate rewards. Patients are guided towards engaging non-eating-disorder rewards that elicit similar positive emotions (e.g., working on a pride-inducing art project) when eating-disorder urges emerge. Counter-conditioning techniques are also used to increase positive emotions in eating-disorder-relevant situations typically associated with negative emotions (e.g., eating a meal) by pairing these situations with rewards (e.g., talking to a friend during the meal).

A second goal is to decrease the reward salience of eating-disorder behaviors. Patients are introduced the metaphor of “riding the rollercoaster” of positive mood to understand the transience of emotion. Patients practice “riding out” dips in positive emotion without engaging in eating-disorder behaviors. Further, the concept of breaking positive emotion links with eating-disorder behavior is also introduced, in which patients are encouraged to engage in behavior that proactively breaks the positive emotion to eating disorder links (e.g., deleting a calorie counting app, removing underweight pictures from social media).

## Module 6: relapse prevention

### Standard PAT

The final session of PAT serves as an opportunity to discuss treatment progress and review future strategies for increasing positive affect in order to avoid relapse.

### Proposed adaptions for AN

In PAT-AN, further discussion may need to be placed on how patients can use PAT-AN skills to continue to reduce eating-disorder thoughts and behaviors. Because sub-threshold eating-disorder cognitions and behaviors continue to persist following treatment [71] and relapse rates are high in AN [72], it may be helpful for the therapist to normalize symptom fluctuations as signals to use PAT-AN skills, while also encouraging the patient to seek treatment when they are having trouble getting “back on track.”

## Potential benefits, pitfalls, and future directions of PAT-AN

There are many aspects of PAT that make it a compelling possible treatment for AN. Most strikingly, an approach in which positive affect and reward are primary treatment targets is entirely unique in eating-disorder treatment [8]. The existing literature demonstrating the existence of transdiagnostic reward-based targets and the positive outcomes of PAT for mood and anxiety suggest the potential for PAT-AN to address both AN and common comorbid conditions [73]. Positive affect has an effect on motivational behavior [74] and, therefore, may enhance the treatment motivation that is often lacking in AN [75] and reduce burnout potential among providers [76]. There are also several hypothesized limitations. Placing a primary focus on targeting positive affect may preclude focus on other important targets, including eating-disorder behavior change, or other putative mechanisms of AN, such as negative affect and habit [77, 78]. Certain aspects of the PAT model, particularly those that focus on experiential exercises, may be difficult for those with a more concrete or pragmatic worldview, such as is often described among individuals with AN [64]. Finally, a 20-session treatment length may not be sufficient to achieve symptom remission, especially for individuals with more severe and enduring AN [2]. However, until further data are available about the efficacy of PAT-AN, the potential benefits and pitfalls of the treatment remain hypothetical.

PAT-AN is in nascent stages of development; therefore, research is needed to determine the efficacy of this intervention for AN. Randomized, controlled trials comparing PAT-AN to treatment as usual or other interventions focused on altering eating-disorder symptoms or reward mechanisms will be a first step in understanding the utility of this treatment for AN [59, 63, 79]. Because PAT-AN is intended as a neuroscience-informed treatment, investigating effects of this treatment on putative behavioral and biological maintaining mechanisms of AN (e.g., frontostriatal reward circuitry and reduce frontoparietal control circuit function) may also be informative. Additionally, the initial test of PAT-AN is being conducted among outpatients with AN. Therefore, even following this trial, further information would be needed to determine if PAT-AN could be effective among populations with differing illness presentations (e.g., specific AN subtypes, BMI severities, and stages of illness) or in different treatment settings (e.g., inpatient). Further, it would be clinically useful to determine if PAT-AN can function as a standalone treatment, or how it could be incorporated as an adjunctive treatment in combination with other disorder-focused interventions (e.g., weight monitoring, meal support).

## Conclusion

Many individuals who receive treatment for AN do not recover [2]. Therefore, the development and adaptation of innovative, alternative intervention approaches is critical. Recent treatment innovations within the anxiety and mood disorders fields [11] provide an optimal foundation from which to develop novel treatments that target reward-based mechanisms that have been long understudied and rarely addressed in AN treatment. Noting prior research implicating aberrant reward-based processing in individuals with AN, targeting positive affect and reward mechanisms via PAT-AN presents an apt, cutting-edge approach to treating this serious and pernicious disorder.

## Data Availability

Not applicable.

## Abbreviations

AN: Anorexia Nervosa
HC: Healthy Comparison
PAT: Positive Affect Treatment
PAT-AN: Positive Affect Treatment for Anorexia Nervosa
